# miR-193a-3p interaction with HMGB1 downregulates human endothelial cell proliferation and migration

**DOI:** 10.1038/srep44137

**Published:** 2017-03-09

**Authors:** Cheen P. Khoo, Maria G. Roubelakis, Jack B. Schrader, Grigorios Tsaknakis, Rebecca Konietzny, Benedikt Kessler, Adrian L. Harris, Suzanne M. Watt

**Affiliations:** 1 Stem Cell Research, Nuffield Division of Clinical Laboratory Sciences, Radcliffe Department of Medicine, University of Oxford, Oxford, OX3 9BQ, UK; 2 Stem Cell Research, NHS Blood and Transplant, Oxford, OX3 9BQ, UK; 3 Laboratory of Biology, National and Kapodistrian University of Athens Medical School, Athens 115 27, Greece; 4 Cell and Gene Therapy Laboratory, Biomedical Research Foundation of the Academy of Athens (BRFAA), Athens, 11527, Greece; 5 Department of Biology, University of York, York, YO10 5DD, UK; 6 Institute of Molecular Biology and Biotechnology, Foundation of Research & Technology, GR-70013 Heraklion, Crete; 7 Target Discovery Institute, NDM Research Building, Nuffield Department of Medicine, University of Oxford, OX3 7FZ, UK; 8 The Weatherall Institute of Molecular Medicine, University of Oxford, John Radcliffe Hospital, Oxford OX3 9DS, UK

## Abstract

Circulating endothelial colony forming cells (ECFCs) contribute to vascular repair where they are a target for therapy. Since ECFC proliferative potential is increased in cord versus peripheral blood and to define regulatory factors controlling this proliferation, we compared the miRNA profiles of cord blood and peripheral blood ECFC-derived cells. Of the top 25 differentially regulated miRNAs selected, 22 were more highly expressed in peripheral blood ECFC-derived cells. After validating candidate miRNAs by q-RT-PCR, we selected miR-193a-3p for further investigation. The miR-193a-3p mimic reduced cord blood ECFC-derived cell proliferation, migration and vascular tubule formation, while the miR-193a-3p inhibitor significantly enhanced these parameters in peripheral blood ECFC-derived cells. Using *in silico* miRNA target database analyses combined with proteome arrays and luciferase reporter assays of miR-193a-3p mimic treated cord blood ECFC-derived cells, we identified 2 novel miR-193a-3p targets, the high mobility group box-1 (HMGB1) and the hypoxia upregulated-1 (HYOU1) gene products. HMGB1 silencing in cord blood ECFC-derived cells confirmed its role in regulating vascular function. Thus, we show, for the first time, that miR-193a-3p negatively regulates human ECFC vasculo/angiogenesis and propose that antagonising miR-193a-3p in less proliferative and less angiogenic ECFC-derived cells will enhance their vasculo/angiogenic function.

Endothelial colony forming cells (ECFCs) or late-outgrowth endothelial cells (OECs) are a subpopulation of endothelial precursors found circulating in human cord blood (CB) and adult peripheral blood (PB), although they are also resident in tissues including the bone marrow, umbilical cord and the placental vasculature^1^,^2^,^3^,^4^. These ECFCs generate *de novo* vessels *in vitro* and in immunodeficient murine models *in vivo,* and their functional potency correlates with their proliferative potential^1^,^2^,^3^,^4^,^5^,^6^,^7^,^8^,^9^,^10^,^11^,^12^,^13^,^14^,^15^,^16^,^17^,^18^,^19^. In pre-clinical models, ECFCs are reported to enhance the patency of implanted vascular grafts and to improve outcomes in ischemic diseases, such as stroke, myocardial infarction and limb ischemia^20^,^21^,^22^,^23^,^24^,^25^,^26^,^27^,^28^,^29^,^30^,^31^. Given their ability to proliferate, migrate, incorporate into vascular networks, release proangiogenic factors and promote vascular repair in preclinical models, ECFCs have attracted substantial interest as a therapeutic target for treating vascular injury.

Studies in non-human primates are reported to recapitulate those in the human, with ECFC frequency and ability to form inosculating vessels decreasing with age^32^. In the human, ECFCs from healthy perinatal CB are significantly more frequent than those found circulating in healthy adult PB and have a higher proliferative capacity^3^,^5^,^6^,^11^,^15^,^16^,^18^,^19^. Thus, defining strategies to enhance the content of these cells in the adult, while retaining their vasculogenic/angiogenic functions would be beneficial. In this respect, there is increasing evidence that small, non-coding microRNAs (miRNAs) regulate endothelial cell generation and functions^33^,^34^,^35^.

miRNAs are short non-coding RNA molecules, 21–25 nucleotides long, that function to downregulate the expression of multiple genes by such processes as mRNA cleavage or repression of translation and acceleration of target mRNA deadenylation. Interestingly, miRNA mediated knockdown impairs vasculogenesis and angiogenesis *in vitro* and in *in vivo* models^36^,^37^,^38^, while miRNA profiling and functional studies have identified both pro-and anti-angiogenic miRNAs^33^,^34^,^37^. Examples of those reported to be negative regulators of angiogenesis include the miR-17, miR-92a, miR-200, miR-221/222, and the 14q32 miR cluster (miR-329, -487b, -494, -495)^34^,^36^,^38^,^39^. In contrast, pro-angiogenic miRNAs include mir-126, let-7f, miR-93, miR-210, miR-130a and miR-13^34^,^38^.

In order to identify miRNAs, which regulate the proliferation of ECFC-derived cells, whilst also enhancing their angiogenic or vasculogenic potential, we compared the microRNA profiles of the more proliferative CB with the less proliferative PB ECFC-derived cells. In this paper, we identify miR193a-3p as an anti-proliferative and anti-angiogenic miRNA, which is preferentially expressed in human adult PB ECFC-derived cells. Subsequently, using miR-193a-3p inhibitors, we demonstrate that these PB cells exhibit improved proliferative and vasculo-/angio-genic abilities compared to control transfected cells. Based on reporter assays, we report that miR-193a-3p inhibits ECFC-derived cell proliferation and subsequently angiogenic functions via a novel target, HMGB1.

## Materials and Methods

### Human endothelial cell culture

Human CB units were sourced from the John Radcliffe Hospital in Oxford with ethical approval from the Oxfordshire Research Ethics Committee C and Berkshire Research Ethics Committee and with informed written consent^6^,^11^. The research was carried out with institutional R&D committee approval, and the University of Oxford and NHSBT Oxford performed all methods in accordance with the relevant guidelines and regulations set. Adult PB cells were obtained from anonymised consented donors and supplied by NHS Blood and Transplant from human leukoreduction filters collected in transparent cones within 4 hr of donation. Nine CB and 9 PB donations were collected and used for the experimental studies. Human ECFC-derived cells were next generated by culturing the CB and PB mononuclear cells (2 × 10^7^ cells in 6 well collagen coated plates) in 4 mls complete EGM-2 media (Lonza Biologics, Cambridge, England) supplemented with 10% (v/v) Hyclone heat inactivated fetal bovine serum (FBS; ThermoScientific, Waltham, MA, USA)^5^,^10^. Endothelial colonies, which formed by 21 days of culture, were selected using cloning rings and those from individual CB or PB units were pooled and then passaged in the above medium^5^,^10^. Passage 0 was the time of the appearance of the first ECFC-derived colonies. The cells possessed a typical endothelial morphology and were confirmed by flow cytometric phenotyping as endothelial lineage cells (Supplemental Methods and Supplementary Fig. S1).

### miRNA microarray analysis

Total and miRNA enriched RNA was extracted using Qiagen RNeasy Mini Kit (Qiagen, Hilden, Germany) according to the manufacturer’s instructions. RNA quality and abundance were determined after extraction using a Nanodrop ND-1000 spectrophotometer (Nanodrop Technologies, Wilmington, NC, USA). miRNA profiling was done by Exiqon A/S, Vedbaek Denmark using 3 individual donations for both CB and PB ECFC-derived cells at passage 3. The samples were labeled using the miRCURY Hy3/Hy5 Power labeling kit and hybridized on the miRCURY LNA Array (version 5^th^ Generation arrays, hsa, mmu and rno)(Exiqon A/S), which consisted of control probes, mismatch probes and 1900 capture probes that perfectly matched all of the human miRNAs registered and annotated in miRBase v15.0. The quantified signals were normalized using the global Lowess (LOcally WEighted Scatterplot Smoothing) regression algorithm, which minimizes the intensity-dependent differences between the dyes. The expression matrix contained normalized Hy3/Hy5 ratios (log2 transformed) from all hybridizations. The subset of differentially expressed miRNAs was used to construct an unsupervised hierarchical clustering of the different samples (Exiqon A/S).

### Reverse transcription PCR and real time-quantitative-PCR (miRNA)

Detailed protocols are provided in the Supplemental information.

### Reverse transcription PCR and real time-quantitative-PCR (mRNA)

Detailed protocols are provided in the Supplemental information.

### miRNA Target Prediction

Bioinformatics prediction of target genes and miRNA binding sites was performed using a combination of TargetScan version 5.1 (http://www.targetscan.com), miRanda August 2010 release (http://www.microrna.org/), mirDB April 2009 (www.mirdb.org), RNA hybrid Version 2.1 (http://bibiserv.techfak.uni-bielefeld.de/rnahybrid/) and miRWalk 2.0 (http://www.umm.uni-heidelberg.de/apps/zmf/mirwalk/).

### miRNA mimic and inhibitor transfections

CB and PB ECFC-derived cells (1 × 10^5^ cells) were plated onto 6 well plates in complete EGM-2 media (Lonza Biologics) overnight. The following day, media were removed and cells transfected with 10 nM mimic non-targeting control, and the relevant miRNA mimic or 10 and 50 nM inhibitor non-targeting control and the relevant miRNA inhibitor (Dharmacon, Lafayette, CO, USA) using Oligofectamine transfection reagent according the manufacturer’s protocol (Life Technologies). After 5 hr transfection, the transfection reagent was replaced with complete EGM-2 media (Lonza Biologics). Cells were harvested for other experiments at 48 hr after transfection.

### siRNA transfection

CB ECFC-derived cells were cultured either at 500 cells/well in 96 well plates, or 0.5 × 10^5^ cells in 12 well plates, and transfected with siRNAs at final concentrations of 5 nM using 0.2% Lipofectamine 2000 (Life Technologies). Two Silencer Select siRNAs (Life Technologies) for HMGB1 (s20254 and s20255) and HYOU1 (s20632 and s20634) were used, together with the siRNA negative control (Silencer Select Negative Control No. 2 siRNA).

### Cell cycle analysis

After 48 hr transfection with miR-193a-3p, miR-34a or control mimics, CB ECFC-derived cells were stained with propidium iodide. The cells were fixed in ice-cold 70% ethanol for at least 16 hr at 4 °C. After fixation, the cells were incubated with 2 mg/ml RNase A (Sigma-Aldrich Ltd. Gillingham, Dorset, England) at 37 °C for 40 minutes. Then, 50 μg/ml propidium iodide (Sigma-Aldrich Ltd.) was added, and the cells were analyzed by flow cytometry. Single cells were selected using pulse area versus pulse height and obvious debris excluded by forward and side scatter gating of single cells, prior to propidium iodide based cell cycle analyses. The data are presented as the mean ± SEM of at least three independent experiments.

### CyQUANT NF proliferation assay

To assess the proliferation of CB and PB ECFC-derived cells, the CyQUANT NF assay (Invitrogen Ltd., Paisley, Scotland) was used. Three to five different CB ECFC-derived cell batches were plated at 5 × 10^2^ cells per well in EGM-2 media in 96 well black walled clear bottomed plates (Corning Life Sciences, Amsterdam, The Netherlands) and incubated overnight at 37 °C in 5% CO_2_. The following day, cells were transfected with miRNA mimics or miRNA inhibitors in parallel with respective mimic non-targeting/negative control or inhibitor non-targeting/negative control miRNAs (n = 5). Subsequently, the CyQUANT NF assay was performed on the same cells on a different plate to obtain fluorescence intensity values for Day 0 (on day of transfection). Cells were incubated with x1 CyQUANT NF dye binding solution for 60 mins at 37 °C and the fluorescence intensity was measured at excitation wavelength of 485 nm and emission detection at 530 nm using VICTOR fluorescence microplate reader (Perkin Elmer, Vienna, Austria). Supernatants from transfected cells were removed after 24 hr and replaced with fresh 10% FBS containing EGM-2 media. At Day 3, the CyQUANT NF assay was performed to obtain fluorescence intensity for Day 3. The proliferation index was calculated using the following formula: (Absorbance value Day 3/Absorbance value Day 0).

### Matrigel and 3D co-culture vascular tubule assays

We used both the matrigel and co-culture assays to assess the effects of miRs on *in vitro* tubule formation by ECFC-derived cells. For the matrigel assay, CB and PB ECFC-derived cells were trypsinized 48 hr after transfection and 1.5 × 10^4^ cells resuspended in complete EGM-2 media in a 96 well plate coated with 50 μl of Growth Factor Reduced Matrigel (GFR matrigel; BD Biosciences, Sunnyvale, CA, USA)^40^. Plates were incubated for 20 hr at 37 °C before photomicroscopy. Each image from each well was taken at x4 magnification using a Nikon Eclipse TE2000-U microscope (Nikon Ltd., London, England). Total tubule length was quantified using Wimasis Image Analysis online platform (www.wimasis.com)^40^.

For the tubule assay, unlabeled human bone marrow mesenchymal stromal cells (hBM MSCs) were co-cultured with transfected CB ECFC-derived cells (transduced with eGFP) in complete EGM-2 media containing VEGF and FGF-2 for up to 14 days as described previously^11^,^18^. For a standard assay, 1.6 × 10^3^ CB ECFC-derived cells or HUVECs were co-cultured with 6.4 × 10^3^ hBM MSCs on a 48-well plate in triplicate wells in complete EGM-2 media (Lonza Biologics). Media were changed every 3–4 days for the duration of the assay. Quantification of the resulting tubules was performed by dividing each well into quarters and photographing eGFP-expressing tubules in each quarter at x4 magnification on a Nikon Inverted TE300 microscope (Nikon UK Ltd.) fitted with a cooled CCD camera and IPLab v3.61 imaging software (Scanalytics; BD Biosciences). Images were processed in Adobe Photoshop 7 and tubule numbers, lengths, and number of junctions were quantified using Angiosys software (TCS Cellworks, Abingdon, England).

### Transwell migration assay

CB and PB ECFC-derived cell migration was evaluated using a modified Boyden chamber assay^15^. Briefly, CB and PB ECFC-derived cells were detached using trypsin/EDTA, harvested by centrifugation, resuspended in EBM-2 media containing 0.5% FBS and counted. Then, 1 × 10^4^ ECFC-derived cells in EBM-2 media containing 0.5% FBS were placed in the upper chamber of a modified Boyden chamber (Corning Life Sciences, Amsterdam, The Netherlands). Complete EGM-2 media were placed in the lower compartment of the chamber. After 4 hrs incubation at 37 °C, the lower side of the filter was washed with PBS and fixed with 4% paraformaldehyde. For visualisation under the fluorescence microscope, cells were stained with DAPI in mounting media (Life Technologies). Cells that migrated into the lower chamber were counted manually in four random microscopic fields (×4).

### Luciferase assay

The pMirTarget-HMGB1-3′-UTR and pMirTarget-HYOU1-3′-UTR were obtained from Origene Technologies Inc. (Rockville, MD, USA). Transient transfections were performed where ~2 × 10^4^ cells were plated in 96-well plates in alpha MEM containing 10% FBS with 1% penicillin-streptomycin 24 hr before transfection. Each vector, along with 10 nM of miR-193a-3p mimic or mimic non-targeting control (Dharmacon), was transfected into HEK293 cells with Lipofectamine 2000 (Invitrogen Ltd.) in Opti-mem media in a 96 well plates, according to the manufacturer’s instructions. Approximately 4–5 hr after transfection, alpha MEM containing 10% FBS with 1% penicillin streptomycin was added. Complete media were changed after 15 hr and incubated for an additional 24 hr, after which luciferase activity was assayed with the Dual-Glo Luciferase Reporter Assay System (Promega, Madison, WI, USA). The pMiRTarget luciferase reporter vector also encodes red fluorescent protein, which was used as a reporter for transfection monitoring and normalization. Relative luciferase activity (firefly luciferase/red fluorescent protein) from the cells transfected with miRNA mimic control was set to 1.0 as a reference. Each experiment was performed at least three times and in triplicate.

### Western blot

Detailed protocols are provided in the Supplementary information.

### Proteome array

Detailed protocols are provided in the Supplementary information.

### Statistics

Experiments were repeated on at least three independent occasions or with at least three biological replicates. Data are presented as the mean ± S.E.M and analysed using unpaired two tailed Student’s t-test, or ANOVA with Dunnett’s post-hoc test or Sidak’s adjustment for multiple comparisons where appropriate, using Prism software version 7.0 (GraphPad software). Differences were considered significant where *p* ≤ 0.05 represented as *p ≤ 0.01 represented as ** and *p* ≤ 0.001 represented as ***.

## Results

### Differential miRNA expression between CB and PB ECFC-derived cells

**Figure 1:**
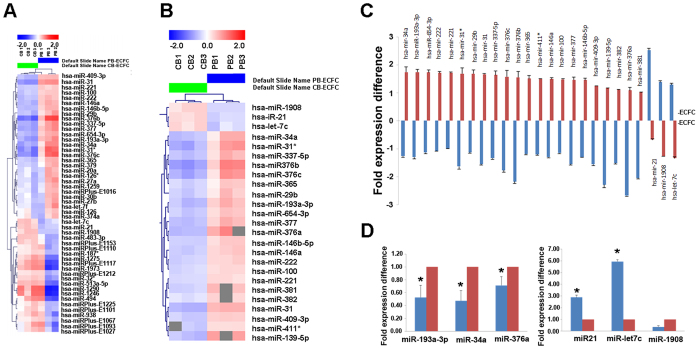
Differentially expressed microRNAs between CB and PB ECFC-derived cells. (**A**) A heat map of two-way clustering of miRNAs and six samples (CB ECFC-derived cells (n = 3) and PB ECFC-derived cells (n = 3)). The clustering was performed on all samples and on all microRNAs on log2 (Hy3/Hy5) ratios, which passed the filtering criteria on variation across samples. Each row represents one microRNA and each column represents one sample. The colour scale shown at the top illustrates the relative expression level of a microRNA across all samples: red colour represents an expression level above mean, blue colour represents expression level lower than the mean. (**B**) A heat map of 25 differentially regulated miRNAs between six different batches of CB ECFC-derived cells (n = 3) and PB ECFC-derived cells (n = 3). These miRNAs were identified based on two-tailed t-test calculated between the two sample groups with p-values < 0.01 in all donor samples. (**C**) Top 25 differentially regulated miRNAs plotted against fold expression difference values calculated for each group versus the pool of samples. MicroRNA’s miR-1908, miR-21 and let-7c were upregulated in CB ECFC-derived cells whilst miR-382, miR-221, miR-100, miR-365, miR-411*, miR-222, miR-29b, miR-146b-5p, miR-409-3p, miR-146a, miR-654-3p, miR-381, miR-337-5p, miR-34a, miR-377, miR-193a-3p, miR-31, miR-139-5p, miR-31*, miR-376c, miR-376a and miR-376b were upregulated in PB ECFC-derived cells. (**D**) q-RT-PCR validation of three miRNAs upregulated in PB ECFC-derived cells (hsa-miR-193a-3p, hsa-miR-34a and has-miR-376a) and three miRNAs upregulated in CB ECFC-derived cells (hsa-miR-21, hsa-let-7c and hsa-miR-1908) normalised to the RNU44 control (*p < 0.05; one-way ANOVA, Sidak’s multiple comparison).

The miRNA profiling of CB and PB ECFC-derived cells identified 50 of 1273 miRNAs that passed the filtering criteria on variation across samples; standard deviation top 50 (Fig. 1A). Further statistical analysis identified twenty five miRNAs that were differentially expressed between CB and PB ECFC-derived cells and are represented in the heat map plot and graph (p < 0.01; Student’s two-tailed t-test; Fig. 1B and C). Three donors per ECFC source were used for miRNA profiling, however we further validated the expression of differentially regulated miRNAs identified in the miRCURY™ LNA Array using an additional 6 different donors of CB and 6 of PB ECFC-derived cells by real-time q-RT-PCR analysis. Three miRNAs (hsa-miR-193a-3p, hsa-miR-34a, hsa-miR-376a) upregulated in PB ECFC-derived cells from all donors tested were chosen for validation by real time q-RT-PCR based on preliminary screen of anti-proliferative activity using mimics. Three additional miRNAs (hsa-miR-21, hsa-let-7c and hsa-miR-1908) chosen from the original miRNA screen as upregulated miRNAs in CB ECFC-derived cells were also used for q-RT-PCR validation. Our results confirmed statistically significant differences between the CB and PB cells for all except one of these miRNAs, hsa-miR-1908. As illustrated in Fig. 1D, for hsa-miR-193a-3p, hsa-miR-34a, hsa-miR-376a, CB ECFC-derived cells showed reduced expression of these miRNAs (0.50 ± 0.03; 0.51 ± 0.02 and 0.39 ± 0.004 fold respectively; p < 0.01, one-way ANOVA) compared to PB ECFC-derived cells (normalized to 1). For hsa-miR-21 and hsa-let-7c, CB ECFC-derived cells showed increased expression of these miRNAs (2.06 ± 0.61; 5.24 ± 1.01 fold respectively; p < 0.05, one-way ANOVA) compared to PB ECFC-derived cells (normalized to 1).

### miR193a-3p limits CB ECFC-derived cell proliferation and cell cycle progression

**Figure 2:**
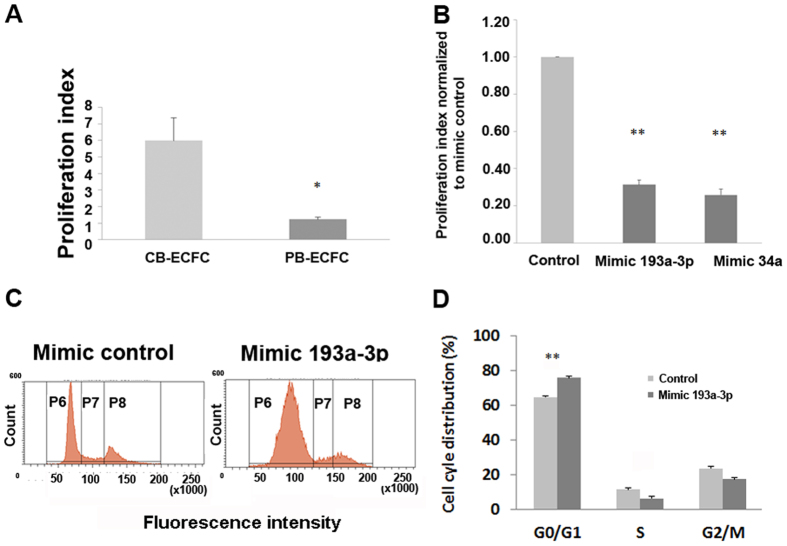
Comparison of CB and PB ECFC-derived cell proliferation. (**A**) CB ECFC-derived cells can proliferate approximately 5–6 times more than PB ECFC-derived cells. Proliferation was measured by the CyQuant assay in which the proliferation index was calculated based on fluorescence values at 72 hr normalised to those obtained for each sample on the day of transfection (0 hr). (**B**) Screen of 2 microRNAs chosen from the microRNA array where CB ECFC-derived cell proliferation was reduced when transfected with 10 nM of miR-193a-3p or miR-34a mimics. Proliferation was measured by the CyQuant proliferation assay at 72 hr post-transfection and data presented as proliferation index normalised to mimic control. (**C**) Effect of miR-193a-3p and control mimics on CB ECFC-derived cell cycle were assayed using propidium iodide by flow cytometry. CB ECFC-derived cells were transfected with miR-193a-3p or control mimics for 48 hr, fixed in 70% ethanol and stained with propidium iodide (Cell cycle stages; p6 = G0/G1; p7 = S and p8 = G2/M). (**D**) Cell percentages from flow cytometry represented in bar chart where overexpression of miR-193a-3p resulted in G1/S cell cycle arrest. The values presented are the mean ± S.E.M of three independent experiments (*p < 0.05; **p < 0.01; one-way ANOVA, Sidak’s multiple comparison).

We have previously isolated and demonstrated the difference in functional capacity of adult PB and CB ECFC-derived cells^5^. These cells have similar cell surface phenotypes as determined by the FACS analyses and by their morphologies (Supplementary Fig. S1); however they differ in their proliferative ability. Notably, PB ECFC-derived cells were on average about 6 times less proliferative in our studies compared to CB ECFC-derived cells as measured by the CyQuant assay (p < 0.05; Student’s t-test; Fig. 2A).

Next, we determined which microRNAs of those differentially regulated in the miRNA arrays for CB compared to PB ECFC-derived cells affected cell proliferation. Initially, we screened 2 different batches of CB ECFC-derived cells transfected with 23 mimics/inhibitors based on those identified in Fig. 1C but excluding hsa-miR411* and hsa-miR-31*, then rescreened 8 of these mimics on CB ECFC-derived cells from 5 different donors and measured their proliferation rate 72 hr post-transfection (data not shown). From these screens, we found that, compared to the control mimic, miR-193a-3p and miR-34a mimics consistently reduced the proliferation of CB ECFC-derived cells (proliferation index compared to the normalized control mimic = 0.31 ± 0.02 and 0.26 ± 0.03 respectively; p < 0.01; one-way ANOVA; Fig. 2B). Transfection efficiency was confirmed using fluorescein tagged mimics (see Supplementary Fig. S2).

Concentrating on miR-193a-3p, and to gain insight into the mechanisms underlying the inhibition of proliferation by miR-193a-3p on human CB ECFC-derived cells, cell cycle analysis was performed (Fig. 2C and D). After transfection of the miR-193a-3p mimic into CB ECFC-derived cells, G1/S arrest was observed. miR-193a-3p overexpression significantly increased the proportion of cells in the G0/G1 phase (75.87% ± 2.92%; p < 0.01; one-way ANOVA) compared with negative control mimic transduced cells (G0/G1: 64.60% ± 2.49%).

### miR-193a-3p reduces CB ECFC-derived cell vascular tubule formation and cell migration

**Figure 3:**
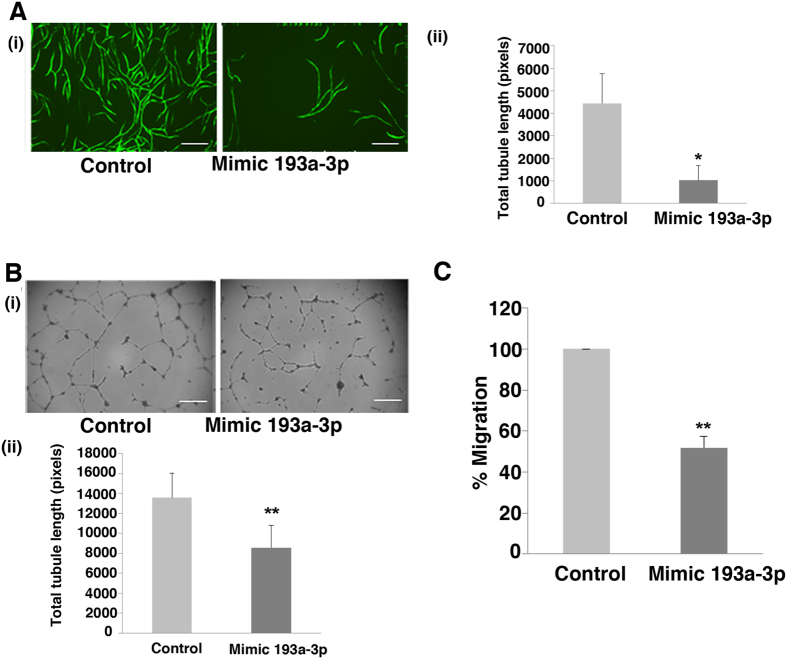
mir-193a-3p effect on CB ECFC-derived cell angiogenic functions. (**A**) Transfected CB ECFC-derived cells were plated on hBM MSCs in a 96 well plate and images at x10 magnification were taken at Day 12 (endpoint). Effect of miR-193a-3p mimic on tubule formation of CB ECFC-derived cells were quantified using Angiosys software (n = 3, *p < 0.05). Scale bar, 100 μm. (**B**) Transfected CB ECFC-derived cells were plated onto growth factor reduced matrigel and allowed to migrate for 18 hr before images at x4 magnification were taken and quantified using Angiosys software (n = 3, p < 0.01). (**C**) CB ECFC-derived cells transfected with 10 nM miR-193a-3p mimic for 48 hr were suspended in 0.5% FBS in EBM-2 and placed in 8 μm transwells and allowed to migrate towards 10% FBS in EGM-2 growth media (**p < 0.01). The values presented are the mean ± S.E.M of three independent experiments (*p < 0.05; **p < 0.01; Student’s t-test).

Next, we set out to determine if miR-193a-3p had an effect on ECFC-derived cell migration and vascular tubule formation. We treated 3 batches of CB ECFC-derived cells with control or miR-193a-3p mimic and confirmed that the latter reduced cell proliferation (Fig. 2B; p < 0.05; Student’s t-test). To assess the effect of this miR-193a-3p mimic on vascular tubule formation, we used an *in vitro* sandwich model, which involved co-culture of hBM MSC and CB ECFC-derived cells (Fig. 3A), and a matrigel assay with CB ECFC-derived cells alone (Fig. 3B). CB ECFC-derived cells transfected with miR-193a-3p mimic demonstrated a significant reduction in total tubule length in the co-culture (Fig. 3A; p < 0.05; Student’s t-test) and matrigel assays (Fig. 3B; p < 0.01; Student’s t-test) compared to the negative control mimic transfected cells. Statistically significant reductions in the numbers of junctions and tubules were also observed with the miR-193a-3p mimic when compared to the negative control mimic. In the vascular co-culture assay (Fig. 3A), the respective number of junctions and number of tubules were 86.1 ± 19.6 vs 8.1 ± 13.2 and 204.2 ± 51.9 vs 34.3 ± 19.8 for the negative control mimic vs miR-193a-3p mimic treatments (p < 0.05 for both; Student’s t-test). In the Matrigel CB-ECFC derived cell assay (Fig. 3B), the respective number of junctions and number of tubules were 83.7 ± 25.5 vs 34.8 ± 17.2 and 175.7 ± 49.7 vs 94.2 ± 31.6 for the negative control mimic vs miR-193a-3p mimic treatments (p < 0.05 for both; Student’s t-test). Furthermore, we observed around a 50% reduced migratory ability in the transwell assay (Fig. 3C) when miR193a-3p mimic was compared to CB ECFC-derived cells transfected with the negative control mimic (p < 0.01; Student’s t-test). These observations indicate that miR-193a-3p is not only an anti-proliferative miRNA, but also an anti-angiogenic/vasculogenic miRNA.

### miR-193a-3p inhibitor improves PB ECFC-derived cell proliferative, migratory and vascular tubule formation ability *in vitro*

**Figure 4:**
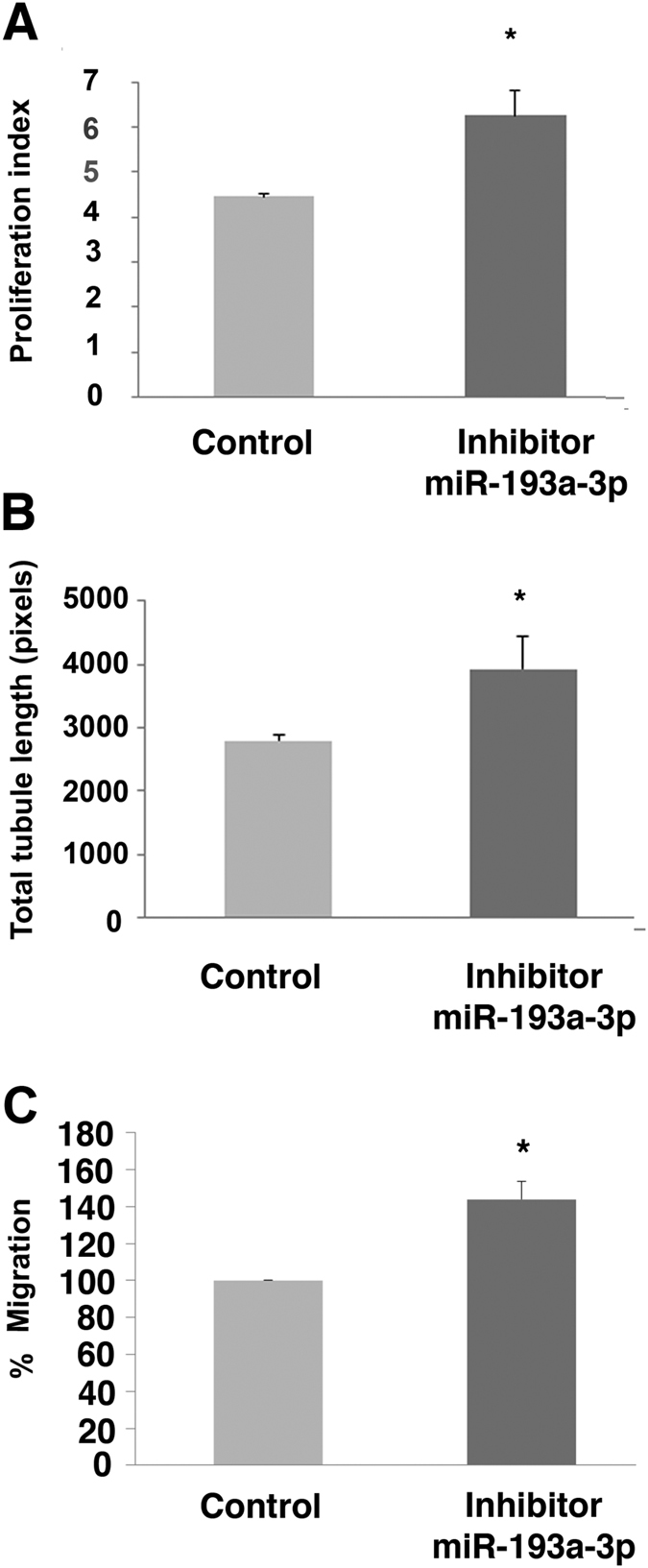
mir-193a-3p inhibitor increases PB ECFC-derived cell proliferation and related angiogenic functions. (**A**) PB ECFC-derived cells transfected with 50 nM miR-193a-3p inhibitor significantly increased cell proliferation compared to cells transfected with inhibitor control (CyQuant assay, n = 3). (**B**) PB ECFC-derived cells transfected with 50 nM miR-193a-3p inhibitor improved tubule formation in the co-culture assay. Effect of miRNA inhibitors on tubule formation of PB ECFC-derived cells was quantified using Angiosys software. (**C**) PB ECtFC-derived cells transfected with 50 nM miR-193a-3p inhibitor improved cell migration towards 10% FBS in EGM-2 growth media. The values presented are the mean ± S.E.M of three independent experiments (*p < 0.05; Student’s t-test).

Based on the anti-angiogenic/vasculogenic action of miR-193a-3p mimic on CB ECFC-derived cells, the effect of a miR193a-3p inhibitor on the relatively less-angiogenic and less proliferative PB ECFC-derived cells was explored. PB ECFC-derived cell numbers increased by 40% (140 ± 9% compared to the control normalized to 100%) over 72 hr post-transfection with 50 nM miR-193a-3p inhibitor, indicating that an inhibition of miR-193a-3p enhances the proliferative potential of adult PB ECFC-derived cells (Fig. 4A; p < 0.05; Student’s t-test). Similarly, miR-193a-3p inhibitor significantly improved tubule formation and cell migration by 41% and 43% respectively (141 ± 7%; 143 ± 10%, respectively) compared to inhibitor control transfected cells (Fig. 4B and C; p < 0.05 for both; Student’s t-test). These results illustrate the potential of the miR-193a-3p inhibitor to not only expand PB ECFC-derived cells in culture but to improve their migratory and vascular tubule formation capabilities.

### HYOU1 and HMGB1 are direct targets of miR-193a-3p

**Figure 5:**
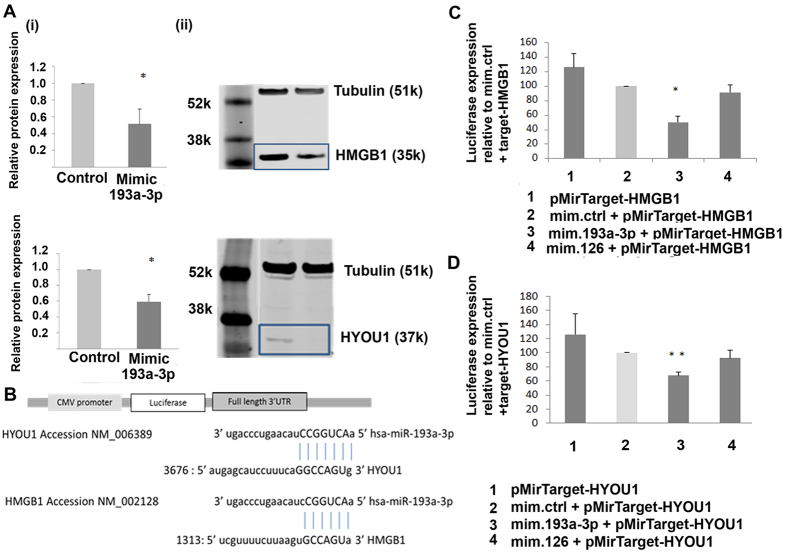
HYOU1 and HMGB1 are direct targets of miR-193a-3p. (**A**) Validation of protein expression of HMGB1 and HYOU1 in control and mimic treated CB ECFC-derived cells using Western blot. The values presented are the mean ± S.E.M of three independent experiments (*p < 0.05; Student’s t-test). Cropped blots were used here and uncropped images of blots are shown in Supplementary Fig. S6. (**B**) Luciferase constructs were used to test whether miR-193a-3p binds to HMGB1 and HYOU1. The 3′ UTRs of HMGB1 and HYOU1 were subcloned into the CMV-driven pMir-target luciferase vector (Origene). (**C**) Luciferase activity of plasmid containing firefly luciferase associated with 3′ region of HMGB1 co-transfected separately with miR-193a-3p mimic (10 nM) was reduced. Co-transfection of HMGB1 with a non-targeted miR-126 did not cause a significant decrease in firefly luciferase activity. Data were normalised to the activity of RFP expressed by the HMGB1 plasmid. (**D**) Luciferase activity of plasmid containing firefly luciferase associated with 3′ region of HYOU1 co-transfected separately with miR-193a-3p mimic (10 nM) was reduced. Co-transfection of HYOU1 with a non-targeted miR-126 did not cause a significant decrease in firefly luciferase activity. Data were normalised to the activity of RFP expressed by the HYOU1 plasmid. The values presented are the mean ± S.E.M of three independent experiments (*p < 0.05; **p < 0.01; one-way ANOVA, Dunnett’s multiple comparison). (Abbreviations: RPP, red fluorescent protein).

In order to understand the mechanism of action of miR-193a-3p on ECFC-derived cell function, mass spectrometry was performed on proteins extracted from non-targeting control and miR-193a-3p mimic treated CB ECFC-derived cells to identify potential targets of miR-193a-3p. Fifty one proteins were discovered to be significantly reduced in all 3 samples of miR-193a-3p mimic treated cells compared to the negative control (Table 1). These proteins were then cross checked using 5 target prediction sites to identify potential binding targets for miR-193a-3p, where 12 were predicted targets of miR-193a-3p (see Supplementary Table S1). HYOU1 and HMGB1 proteins with a fold change of 1.9 and 2.6 respectively (Table 1) were chosen for further investigation based on their known role in proliferation in other cell types, and as they have not been confirmed experimentally to be miR193a-3p targets. qPCR studies demonstrated statistically significant 1.88 and 2.33 fold higher levels of HYOU1 and HMGB1 respectively in CB ECFC-derived cells when compared to PB ECFC-derived cells (Supplementary Fig. S3). To validate the CB proteome array results, CB ECFC-derived cells were treated with miR-193a-3p mimic, and then protein lysates collected for Western blot analysis after 48 hr incubation. The results show that HMGB1 and HYOU1 protein levels were reduced approximately 2 fold in cells transfected with miR-193a-3p mimic compared to the control mimic treated cells (Fig. 5A).

To demonstrate the direct interactions of miR-193a-3p with HMGB1 and HYOU1 3′UTRs harbouring the potential binding site to miR-193a-3p, constructs of these regions were cloned into the pMir-Target plasmid downstream of a luciferase reporter gene (Fig. 5B). A significant decrease of 50% and 32% (to 50 ± 8% and 68 ± 5% respectively of the normalized mimic control) in luciferase activity was observed in cells transfected with pMirTarget-HMGB1 and pMirTarget-HYOU1 constructs respectively when treated with 10 nM miR-193a-3p mimic, relative to the same concentration of a control mimic (Fig. 5C and D; p < 0.05 and p < 0.01 respectively; one-way ANOVA). As an additional control, there was no significant difference between cells co-transfected with the target constructs and miR-126, a negative control miRNA validated to not target either HMGB1 or HYOU1 (Fig. 5C and D; 92 ± 11%; p = 0.28 and 92 ± 9%; p = 0.11; one-way ANOVA). Empty vector controls are shown in Supplementary Fig. S4. This provides strong evidence of a direct molecular binding interaction between miR-193a-3p and the 3′UTRs of HMGB1 and HYOU1, indicating that these genes are novel direct targets of miR-193a-3p.

### HMGB1 siRNA suppresses CB ECFC-derived cell proliferation and migration

**Figure 6:**
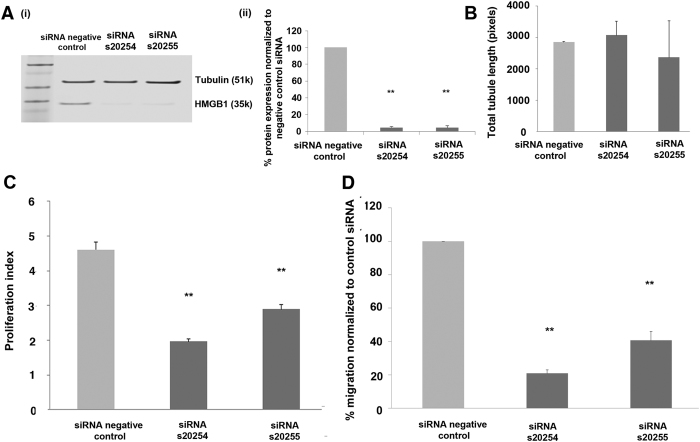
HMGB1 knockdown affects CB ECFC-derived cell proliferation and migratory abilities. (**A**) (i) Western blot of HMGB1 protein in CB ECFC-derived cells following transfection of 2 different siRNAs for HMGB1, each at a final concentration of 5 nM. (ii) Western blot quantification of HMGB1 protein levels where the target protein expression was normalised to tubulin expression in each sample. Results show target gene protein levels as a percentage of the negative control siRNA-transfected sample. Cropped blots were used here and uncropped images of blots are shown in Supplementary Fig. S7. (**B**) No significant effect of HMGB1 siRNA knockdown on the tubule formatting ability of CB ECFC-derived cells. Cells treated with a negative control siRNA, HMGB1 s20254 siRNA, or HMGB1 s20255 siRNA at a final concentration of 5 nM were subjected to a matrigel assay for 18 hr, 48 hr post-transfection. (**C**) The effect of HMGB1 siRNA knockdown on the proliferative and/or survival ability of CB ECFC-derived cells. Cells treated with either a negative control siRNA, HMGB1 s20254 siRNA, or HMGB1 s20255 siRNA at a final concentration of 5 nM were subjected to a CyQuant proliferation assay at 72 hr to measure proliferation. Fluorescence values were normalised to those obtained on the day of transfection for each sample to obtain a proliferation index for each condition. (**D**) The effect of HMGB1 siRNA knockdown on the migration ability of CB ECFC-derived cells. Cells treated with a negative control siRNA, HMGB1 s20254 siRNA, or HMGB1 s20255 siRNA at a final concentration of 5 nM were subjected to a transwell assay for 5 hr towards 10% FBS in EGM-2 growth media following 48 hr post-transfection. The values presented are the mean ± S.E.M of three independent experiments (**p < 0.01; one-way ANOVA, Dunnett’s multiple comparison).

Following the demonstration that HYOU1 and HMGB1 are direct targets of miR-193a-3p, siRNA experiments were carried out to determine whether these targets affect CB ECFC-derived cell angiogenic/vasculogenic functions. CB ECFC-derived cells were transfected with HMGB1 and HYOU1 specific siRNAs. Protein knockdown of approximately 90% was achieved for HMGB1 s20254 and s20255 siRNAs (Fig. 6A; p < 0.01; one-way ANOVA) but only approximately 20–30% for siRNA HYOU1 s20632 and s20634 siRNAs (s20632; p < 0.05; s20634; p < 0.05; one-way ANOVA) (see Supplementary Fig. S5). Since HMGB1 protein was efficiently suppressed using these siRNAs, the functional characteristics of CB ECFC-derived cells upon knockdown of this novel target were examined. HMGB1 siRNAs s20254 and s20255 significantly affected the number of CB ECFC-derived cells generated over 72 hr (Fig. 6C; p < 0.01; one-way ANOVA). Similar results were observed where HMGB1 siRNAs s20254 and s20255 affected CB ECFC-derived cell migration towards 10% EGM-2 growth media containing factors such as VEGF and bFGF (Fig. 6D; p < 0.01; one-way ANOVA). However, HMGB1 siRNAs s20254 and s20255 did not significantly affect tubule formation in the matrigel assay when compared to the control (Fig. 6B; p = 0.87; one-way ANOVA). This indicates that knocking down HMGB1 affects endothelial cell migration towards specific factors and also proliferation but is not necessary for their ability to form vascular tubules in matrigel.

Unlike HMGB1 siRNAs, HYOU1 siRNAs did not affect CB ECFC-derived cell proliferation under the conditions used for cell culture (s20632; p = 0.914; s20634; p = 0.813; one-way ANOVA) nor migration (s20632; p = 0.528; s20634; p = 0.343; one-way ANOVA; Supplementary Fig. S5). These siRNA results demonstrate that HMGB1 plays a role in both CB ECFC-derived cell migratory and proliferative ability, which mimics the same effects of miR-193a-3p on CB ECFC-derived cells shown in Fig. 3.

## Discussion

A number of microRNAs have already been shown to regulate endothelial cell functions, such as cell proliferation, senescence, migration, differentiation and vascular tubule formation^33^,^34^,^37^,^38^,^41^. However, in the past there has been considerable controversy in defining true circulating endothelial lineage progenitor cells. It is now well established that cells which promote revascularization can be identified in the circulation as proangiogenic myeloid/hematopoietic cells (formerly termed early EPCs) and endothelial colony forming cells or ECFCs (formerly termed late EPCs) based on whether they belong to the hematopoietic or endothelial lineages^1^,^42^. Additionally, microRNA analyses comparing circulating peripheral blood proangiogenic cells with umbilical cord ECFC-derived cells (HUVEC) have demonstrated mutually exclusive miRNA profiles^43^,^44^.

We and others have observed that circulating CB and PB ECFC-derived cells exhibit differences in cell proliferation and vascular tubule formation^3^,^5^,^6^,^11^. Therefore, in this study, we investigated differences in the post-transcriptional regulation of circulating CB and PB ECFC-derived cells by comparing their microRNA profiles. We identified 25 microRNAs differentially regulated between CB and PB ECFC-derived cells. Within the 22 miRNAs upregulated in PB ECFC-derived cells, we detected miRNAs with both known anti-angiogenic properties (e.g. miR-221, miR-222 and miR-34a)^33^,^34^,^37^,^45^ or with undefined properties. Of the latter, the miR-193a-3p mimic significantly reduced the proliferative and vasculogenic/angiogenic functions of CB ECFC-derived cells and this miRNA was chosen for further study. Subsequently, miR-193a-3p inhibition in PB ECFC-derived cells resulted in a statistically significant increase in the proliferation rate of these cells. Other studies have examined the role of miR193a (-3p) in tumor cells. For example, in agreement with the effects we observed on cell proliferation, Nakano *et al*.^46^ identified miR-193a as the strongest candidate affecting cell proliferation and death in A2780 ovarian cancer cells after performing a gain-of-function miRNA screen. Similarly, miR-193a(-3p) has been implicated in regulating proliferation of different tumor cell types, for example, by targeting c-kit in acute myeloid leukemia cells^47^, CCND1, ERBB4, Mcl-1, STMN1 and KRAS in ovarian cancer cells^46^ and ERBB4 in lung cancer cells^48^.

To understand the potential mechanisms by which miR-193a-3p exerts its anti-proliferative and anti-angiogenic effect on ECFC-derived cells, we performed proteomic analysis on CB ECFC-derived cells that were treated with and without miR-193a-3p mimics and validated our proteome results by using target prediction analyses and luciferase reporter assays. Consequently, we identified HMGB1 and HYOU1 as potential targets of miR-193a-3p in ECFC-derived cells. More importantly, our siRNA knockdown studies resulted in significant effects on HMGB1 related vasculogenic/angiogenic functions. High-mobility group box-1 (HMGB1) protein is a transcription factor that has a dual role as a mobile chromatin protein and as a cytokine^49^. Amongst its functions, HMGB1 has been implicated in tissue repair and regeneration by promoting chemotaxis of stem/progenitor cells, stimulating the proliferation of tissue resident stem/progenitor cells including endothelial cells and vascular smooth muscle cells, acting as a proangiogenic cytokine and promoting secretion of proangiogenic cytokines such as VEGF and CXCL8^50^,^51^,^52^,^53^,^54^. Interestingly, HMGB1 has been reported to promote endothelial recruitment via integrin activation^50^ and endothelial proliferation, migration and angiogenesis via RAGE and/or TLR4 signalling pathways, thus effecting tissue repair^55^,^56^,^57^,^58^,^59^,^60^,^61^,^62^,^63^. Additionally, recent studies using nuclear magnetic resonance and surface plasmon resonance analyses have demonstrated that upon inflammation HMGB1 can form a heterocomplex with CXCL12 which will then signal via CXCR4^58^. HMGB1 binding to CXCL12 induces changes in residues 3–12 of the latter, which are fundamental for the triggering of its receptor, CXCR4^58^. During the initial phase of tissue injury, the CXCL12–HMGB1 complex has been reported to mediate the recruitment of mononuclear cells, thereby cooperating in promoting cell migration *in vitro* and *in vivo*^58^. We have shown previously that CXCR4-CXCL12 interactions in ECFC-derived cell and mesenchymal stromal cell co-cultures are important in vascular tubule formation^11^, both for endothelial tip-like cell migration and the integration of ECFC-derived cells into developing vascular networks.

Our results have demonstrated for the first time that miR-193a-3p directly suppresses HMGB1 in CB ECFC-derived cells. When HMGB1 is silenced, this affects both vasculogenesis/angiogenesis as shown in both our and other studies^50^,^55^,^56^,^57^,^59^,^60^,^61^,^62^,^63^,^64^. Given this, other molecules identified in our proteomic screen and/or as potential targets for miR193a-3p may also play a role in modulating angiogenesis. Exemplars include firstly ILK (integrin-linked kinase), which has been reported to promote angiogenesis and to be involved in endothelial cell migration, tubule formation and proliferation *in vitro*^65^,^66^,^67^,^68^. Secondly, STMN1 (stathmin) regulates the proliferation, migration, and network formation of cultured endothelial cells as well as angiogenesis *in vivo*^69^. Thirdly, CUL2 (cullin 2) is a scaffold protein critical for the assembly of the ubiquitin ligase system and therefore for Hif-alpha stabilization^70^. Hif-alpha proteins are known to have a role in promoting angiogenesis^71^. Finally, CD73 (NT5E) has been reported to be involved in tumour angiogenesis^72^,^73^,^74^. These molecules warrant further investigation, which is beyond the scope of this current manuscript, as targets for miR193a-3p.

## Conclusion

We have demonstrated that miR-193a-3p regulates the proliferation and the vasculogenic/angiogenic properties of ECFC-derived cells, at least in part, by direct suppression of HMGB1. Therefore, targeting miR-193a-3p in ECFC-derived cells may both enhance the numbers of these cells and improve their vasculo-/angio-genic function.

### Data Access

MicroRNA array files and analysis has been have been deposited in NCBI’s Gene Expression Omnibus^75^ and are accessible through GEO Series accession number GSE81574 (https://www.ncbi.nlm.nih.gov/geo/query/acc.cgi?acc=GSE81574).

## Additional Information

**How to cite this article:** Khoo, C. P. *et al*. miR-193a-3p interaction with HMGB1 downregulates human endothelial cell proliferation and migration. *Sci. Rep.* **7**, 44137; doi: 10.1038/srep44137 (2017).

**Publisher's note:** Springer Nature remains neutral with regard to jurisdictional claims in published maps and institutional affiliations.

## Supplementary Material

Supplementary Information

## Figures and Tables

**Table 1:** Differentially regulated proteins between mimic control and miR-193a-3p mimic CB ECFC-derived transfected cells.

Accession number (UniProt)	Gene symbol	Peptide count	Anova (p)	Fold change	miR-ctrl abundance	miR-193 abundance
Q13418	ILK	1	2.45E-07	66.52	459.04	6.90
P10155	TROVE2	2	3.07E-05	18.14	823.07	45.37
P10768	ESD	2	0.000177465	11.93	2060.45	172.78
O00154	ACOT7	1	9.15E-05	10.34	1220.57	118.02
P35637	FUS	7	7.70E-11	10.11	47744.45	4721.85
P17612	PRKACA	1	9.97E-05	9.87	412.52	41.81
P62304	SNRPE	1	5.34E-06	6.75	6191.37	917.68
P28289	TMOD1	1	0.000119239	5.64	36804.73	6522.19
Q9H6R0	DHX33	1	8.58E-05	5.55	2326.65	419.27
Q9UHY1	NRBP1	1	8.81E-06	5.35	726.59	135.78
Q8IVL6	LEPREL2	2	6.52E-10	5.23	1394.90	266.83
Q8WUM0	NUP133	1	0.003479238	5.20	779.99	149.88
Q9NQW7	XPNPEP1	2	4.97E-06	4.86	2464.51	506.68
Q9H814	PHAX	2	1.29E-05	4.58	354.99	77.47
P21589	NT5E	2	9.55E-06	4.06	3832.37	944.69
Q96HP0	DOCK6	2	2.60E-07	3.87	891.69	230.35
Q16630	CPSF6	2	0.000146707	3.79	2407.28	635.46
Q13283	G3BP1	7	1.23E-07	3.08	22884.07	7419.24
P31943	HNRNPH1	8	3.61E-06	3.08	43520.52	14120.52
Q13144	EIF2B5	1	0.000122997	3.03	396.01	130.75
Q9NZ08	ERAP1	1	9.11E-07	3.03	720.36	237.90
P56134	ATP5J2	3	1.83E-07	2.90	9372.84	3236.49
Q13617	CUL2	3	5.02E-05	2.66	1224.81	459.72
P09429;B2RPK0	HMGB1	9	9.58E-12	2.61	259753.91	99477.90
P0C0S5	H2AFZ	4	5.12E-09	2.57	106311.36	41414.44
P34897;P34896	SHMT1	8	3.80E-12	2.48	39153.06	15776.54
P26583	HMGB2	4	1.08E-07	2.47	6248.59	2526.31
O00629	KPNA4	1	3.88E-05	2.45	1161.75	474.34
Q92900	UPF1	1	6.31E-06	2.17	1053.91	485.88
Q7L2H7	EIF3M	5	7.47E-06	2.09	5984.93	2862.97
P78344	EIF4G2	3	2.72E-07	2.09	2745.60	1316.24
O76099	OR7C1	1	1.39E-05	2.07	14322.84	6931.60
Q9HB71	CACYBP	6	5.84E-05	2.06	27717.15	13463.32
P16949	STMN1	3	6.43E-08	2.04	9322.38	4565.74
P33991	MCM4	1	6.60E-09	2.03	3383.42	1663.71
P63167;Q96FJ2	DYNLL1	2	6.02E-07	1.98	16194.26	8159.44
O14980	XPO1	5	2.23E-06	1.93	9380.00	4853.49
P09211	GSTP1	14	1.33E-06	1.91	180260.98	94165.74
Q9Y4L1	HYOU1	16	4.67E-10	1.91	103604.44	54211.42
P12004	PCNA	9	3.50E-08	1.87	63904.00	34098.36
O95782;O94973	AP2A1	2	9.14E-10	1.83	2036.05	1110.96
Q6PIU2	NCEH1	2	2.55E-09	1.83	5022.86	2747.12
P40227	CCT6A	9	1.86E-06	1.81	26643.10	14703.73
Q15365	PCBP1	6	6.60E-12	1.80	96494.89	53709.70
Q92973;O14787	TNPO1	9	1.57E-05	1.74	38450.21	22089.48
P46778	RPL21	6	2.38E-08	1.72	87866.75	51097.70
P18669;P15259;Q8N0Y7	PGAM1	10	8.96E-07	1.71	153025.36	89602.32
Q1KMD3;Q6ISU1	HNRNPUL2	5	5.88E-08	1.65	11874.20	7177.37
Q06323	PSME1	3	3.69E-07	1.62	11671.43	7218.58
O15145	ARPC3	3	1.72E-06	1.59	22377.31	14076.88
P53801	PTTG1IP	1	2.39E-07	1.56	12013.45	7717.98

Comparison of proteomic profile of mimic control and miR-193a-3p mimic CB ECFC-derived transfected cells. Fifty one differentially regulated proteins between the two conditions were identified and presented based on highest to lowest fold change between mimic control and miR-193a-3p treated samples.
