# Supplementary material for: miR-193a-3p interaction with HMGB1 downregulates human endothelial cell proliferation and migration
Source: Sci Rep. 2017 Mar 9;7:44137. doi: 10.1038/srep44137 (PMC5343468; doi:10.1038/srep44137)
Supplement: Supplementary Information [file srep44137-s1.pdf]

Supplementary Information

miR-193a-3p interaction with HMGB1 downregulates human endothelial cell proliferation and migration

Cheen P. Khoo[1,2], Maria G. Roubelakis[1-4], Jack B. Schrader[1,2,5],Grigorios Tsaknakis[1,2,6], Rebecca Konietzny [7], Benedikt Kessler[7], Adrian L. Harris[8] and Suzanne M. Watt[1,2].

1. Stem Cell Research, Nuffield Division of Clinical Laboratory Sciences, Radcliffe Department of Medicine, University of Oxford, Oxford, OX3 9BQ. UK.
2. Stem Cell Research, NHS Blood and Transplant, Oxford, OX3 9BQ. UK.
3. Laboratory of Biology, National and Kapodistrian University of Athens Medical School, Athens 115 27, Greece.
4. Cell and Gene Therapy Laboratory, Biomedical Research Foundation of the Academy of Athens (BRFAA), Athens, 11527, Greece.
5. Department of Biology, University of York, York, YO10 5DD. UK.
6. Institute of Molecular Biology and Biotechnology,Foundation of Research & Technology, GR-70013 Heraklion, Crete.
7. Target Discovery Institute, NDM Research Building, Nuffield Department of Medicine, University of Oxford, OX3 7FZ. UK.
8. The Weatherall Institute of Molecular Medicine, University of Oxford, John Radcliffe Hospital, Oxford OX3 9DS. UK.

Supplementary Materials and Methods

Umbilical cord blood ECFC-derived cell phenotyping

Three batches of CB and PB ECFC-derived cells at approximately 80% confluency were detached from tissue culture flasks using 100% (v/v) accutase (PAA Laboratories,Yeovil, UK) for 5 min and washed and resuspended in magnetic activated cell sorting (MACS) buffer containing 1.0% (w/v) bovine serum albumin (BSA) and Fc receptor (FcR) blocking agent (Miltenyi Biotech, Bergisch-Gladbag, Germany) at 4^0C prior to labeling with relevant conjugated monoclonal antibodies (Mabs) or isotype-matched negative-controls as described[1]. The following mouse Mabs were used: PE-CD31 (mIgG1; clone WM59), PE-CD73 (clone AD2), PE-CD144 (clone 16B1), PE-CD166 (clone 105902), PE-CD133 (mIgG2b, clone 293C3), PE-mIgG1 isotype controls, FITC-CD105 (mIgG1; clone FAB10971F), FITC-CD146

(clone MAB16985F),, FITC-mIgG1 isotype control, APC-CD34 (mIgG2a, clone AC136), APC-mIgG2a isotype control, PE-Cy7- CD14 (mIgG2a, clone M5E2), PE-Cy7-CD45 (mIgG1, clone 2D1) and PE-Cy7 mIgG1 and mIgG2a isotype controls. Cells were analyzed on a BD LSR II flow cytometer using FACS Diva software (BD Biosciences) as previously described[1]. Cells were stained using single conjugated antibodies and then incubated with 1:1000 DAPI (Molecular Probes, Paisley, Scotland) to select viable cells prior to CD antigen analysis.

Western blot

Western blot was performed with protein extracts from CB and PB ECFC-derived cells transfected with miR-193a-3p mimic and mimic non-targeting control. Cell pellets were lysed on ice for 30 min in lysis buffer containing 20 mM Tris-Cl, 137 mM NaCl, and 10% glycerol with x1 protease inhibitors. The supernatant collected after centrifugation was quantitated, and 50 µg of protein extract was loaded onto a NuPage 4-12% Tris Bis gel (Invitrogen). The following primary mouse antibodies were used: HYOU1 (clone 6F7; Abnova Corporation, Taipei City, Taiwan) and HMGB1 (clone EPR3507; Abcam, Cambridge, UK) for 1 h at room temperature. Respective secondary antibodies were used and detection of positive signal was achieved using Odyssey CLx for Infrared Fluorescent imager plus (Licor Biosciences). Normalization was performed using antibody against alpha tubulin at 1:5000 (BD Biosciences).

Reverse transcription PCR and real time-quantitative-PCR (miRNA)

Isolated RNAs were converted to cDNA using TaqMan MicroRNA Reverse Transcription Kit (Applied Biosystems, Foster City, CA, USA) and 20ng RNA. Mature human microRNA expression of miR-193a-3p, miR-34a, miR-376a, miR-21, let-7c and miR-1908 was determined by real-time qPCR using TaqMan microRNA assays that contains target-specific stem-loop reverse transcription primers (Applied

Biosystems; Assay 002250 for mir-193a-3p; Assay 002316 for miR-34a; Assay 000565 for miR-376a ;Assay 000397 for miR-21; Assay 000379 for let-7c and Assay 121109_mat for miR-1908) and performed on ViiA 7 q-RT-PCR (Life Technologies, Carlsbad, CA, USA). Fold changes in microRNA expression were determined by the $2^{-\Delta\Delta Ct}$ method, normalizing the results to expression of the control RNU 44[2]. Each PCR reaction was carried out in triplicate according to Taqman MicroRNA assay instructions and on a 96-well optical plate performed at 95°C for 10 minutes, followed by 40 cycles of 95°C for 15 seconds, and 60°C for 60 seconds.

Reverse transcription PCR and real time-quantitative-PCR (mRNA)

Isolated RNAs were converted to cDNA using TaqMan using the High-Capacity RNA-to cDNA Kit (Applied Biosystems) using 40ng RNA. mRNA expression of HMGB1 and HYOU1 was determined by real-time q-RT-PCR using TaqMan Gene Expression Assay primers (Life Technologies; HMGB1: Hs01590761_g1, HYOU1: Hs00197328_m1) and performed on ViiA 7 q-RT-PCR (Life Technologies). Fold changes in mRNA expression were determined by the 2-ΔΔCt method, normalizing the results to expression of the control beta-2-microglobulin. Each PCR reaction was carried out in triplicate according to Taqman Gene Expression assay instructions and on a 96-well optical plate performed at 95°C for 10 minutes, followed by 40 cycles of 95°C for 15 seconds, and 60°C for 60 seconds.

Proteome array analysis by mass spectrometry

CB ECFC-derived cells (n=3) were transfected in parallel with mimic non-targeting control (10nM) and miR-193a-3p mimic (10nM) for 48 hr. Cells were washed with cold PBS and centrifuges. Cell pellets were frozen at -80°C before samples were processed by the Target Discovery Institute Mass Spectrometry Laboratory, University of Oxford. Briefly samples were lysed by mixing with 150 µl of solution containing 4% SDS, 100mM Tris/HCl pH 7.6, 0.1M DTT (lysis solution) and

incubation at 95°C. Lysates were clarified by high speed centrifugation and cleaned several times to remove SDS. HPLC-purified peptides were analyzed with nano–liquid chromatography tandem mass spectrometry (nano–LC-MS/MS), using an Acquity LC instrument (C18 column with a 75µmx250mm, 1.7µm particle size; Waters) coupled to a Thermo LTQ Orbitrap Elite mass spectrometer (resolution of 120,000 @ 400 m/z, Top 20, collision-induced dissociation), using a gradient of 1–35% acetonitrile for 60 min at a flow rate of 250 nl/minute. Peak lists containing MS/MS spectra were generated using MSConvert and these lists were then searched, using Mascot version 2.3, against the Swiss-Prot protein database with the taxonomy restriction "mouse" (16,642 entries as of September 2012) or "human" (20,306 entries as of June 2014), with tryptic restriction and with mass deviations of 10 parts per million/0.5 daltons in the respective MS modes. Quantitative and statistical analysis were performed using Progenesis-QI (Non Linear Dynamics) using transformed normalized abundances for one-way analysis of variance (ANOVA). ANOVA values of $P < 0.05$ and additionally regulation of >1.5-fold were regarded as significant.

References

1 Newey, S. E. et al. The hematopoietic chemokine CXCL12 promotes integration of human endothelial colony forming cell-derived cells into immature vessel networks. Stem Cells Dev 23, 2730-2743 (2014).

2. Schmittgen, T. D. & Livak, K. J. Analyzing real-time PCR data by the comparative C(T) method. *Nat Protoc* **3**, 1101-1108 (2008).

Supplementary Figures

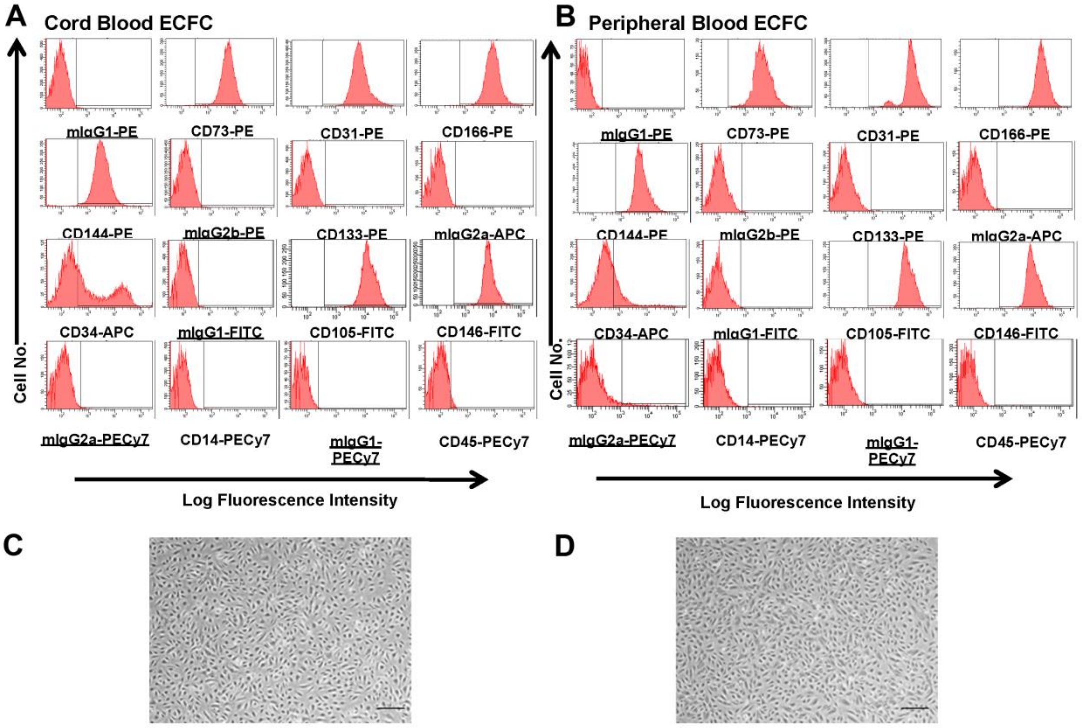

Supplementary Fig. S1. Characterization of CB and PB ECFC-derived cells

A. Representative FACS histograms of CB ECFC-derived cells at passage 4–6.

Cells were 99.8%±0.1% CD73+, 99.3% ±0.7% CD31+, 99.9%±0.04% CD166+ , 95.9%±2.3% CD144+ , 98.7%±0.8% CD105+, and 99.0%±0.9% CD146+, but did not express CD133, CD14, or CD45 (2.0%±0.5% CD133+, 0.6%±0.2% CD14+, and 5.4%±0.4% CD45+. Isotype-negative control values were 1.53% ± 0.4% mIgG1-PE+, 1.12%±0.1% mIgG2b-PE+ , 2.18%± 0.6% mIgG2a-APC+ , 0.83%±0.1% mIgG1-FITC+ , 1.87%±0.2% mIgG2a-PeCy7+ , and 0.62%±0.1% mIgG1-PeCy7+.

B. Representative FACS histograms of PB ECFC-derived cells at passage 4–6.

Values were expressed as mean – standard error of the mean for n = 3 independent batches of cells. Cells were 99.3%±0.3% CD73+, 99.7%±0.1% CD31+, 99.4%±0.3% CD166+ , 99.4%±0.2% CD144+ , 99.6%±0.1% CD105+, and 99.6%±0.1% CD146+, but did not express CD133, CD14, or CD45 (1.4%±0.5% CD133+, 1.9%±1.1%

CD14+, and 0.2%±0.04% CD45+. Isotype-negative control values were 0.55%±0.1% mIgG1-PE+ , 0.63%±0.2% mIgG2b-PE+ , 0.25%±0.01% mIgG2a-APC+, 1.08%±0.3% mIgG1-FITC+, 5.6%±0.8% mIgG2a-PeCy7 , and 0.55%±0.1% mIgG1-PeCy7+.

C. Representative photomicrographs of ECFC-derived cells cultured from cord blood MNCs (×10 magnification, 100μm) of culture at passage 3.

D. Representative photomicrographs of ECFC-derived cells cultured from peripheral blood MNCs (×10 magnification, 100μm) of culture at passage 3.

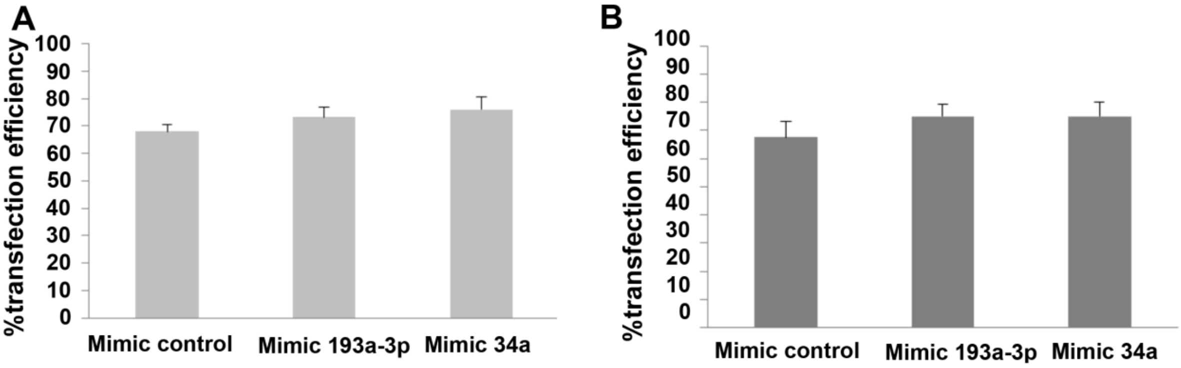

Supplementary Fig. S2. Transfection efficiency of miRNA mimics into CB and PB ECFC-derived cells

10nM miRNA mimics tagged with fluorescein at the 5’ end were used to transfect **A.** PB ECFC-derived cells and **B.** CB ECFC-derived cells. The percentage transfection efficiency were measured using flow cytometry after 48 hr of transfection.

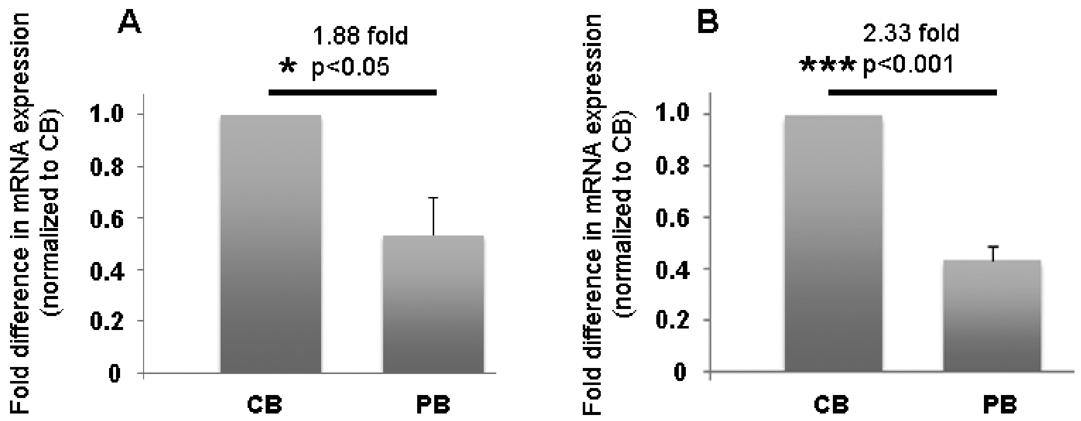

Supplementary Fig. S3: Quantitative PCR (qPCR) analysis comparing mRNA expression of miR-193a-3p HYOU1 and HMGB1 targets in CB and PB ECFC-derived cells.

qRT-PCR was used to quantitate (A) HYOU1 and (B) HMGB1 mRNA expression relative to beta-2-microglobulin expression in CB ECFC-derived cells (n=5) versus PB ECFC-derived cells (n=3). The fold difference in expression was determined by normalizing data to HYOU1 or HMGB1 mRNA expression in CB ECFC-derived cells which was set at 1. Data are presented as the mean of 3 independent experiments ± S.E.M. Statistical analysis is by two tailed unpaired Student's T test where significance values are *p<0.05 and ***p<0.001.

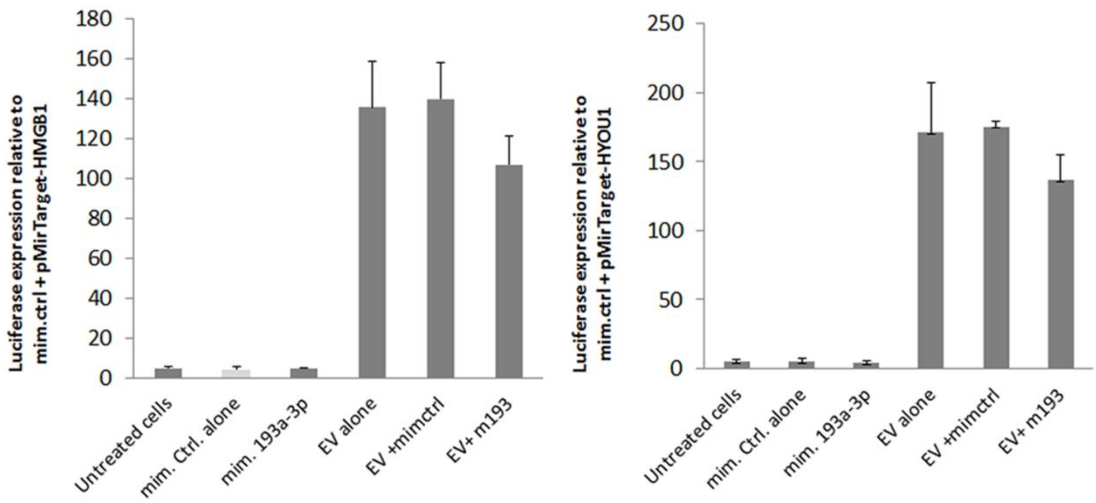

Supplementary Fig. S4. Supporting controls for luciferase HYOU1 and HMGB1 binding experiments

Luciferase activity of plasmid containing firefly luciferase associated with 3’ region of HYOU1 and HMGB1 was investigated and a number of control conditions were used in parallel including cells transfected with i) control mimic, ii) miR-193a-3p mimic, iii) empty pMirTarget (EV), iv) empty pMirTarget (EV) together with control mimic and v) empty pMirTarget (EV) together with miR-193a-3p mimic. As expected, co-transfection of cells with control mimic and miR-193a-3p mimic did not give any firefly luciferase activity. Conversely, EV which contains the luciferase transcript exhibited luciferase activity. Data were normalised to the activity of RFP expressed by HYOU1 or HMGB1 plasmids respectively. The values presented are the mean ± S.E.M of three independent experiments (Abbreviations: EV, empty pMirTarget)

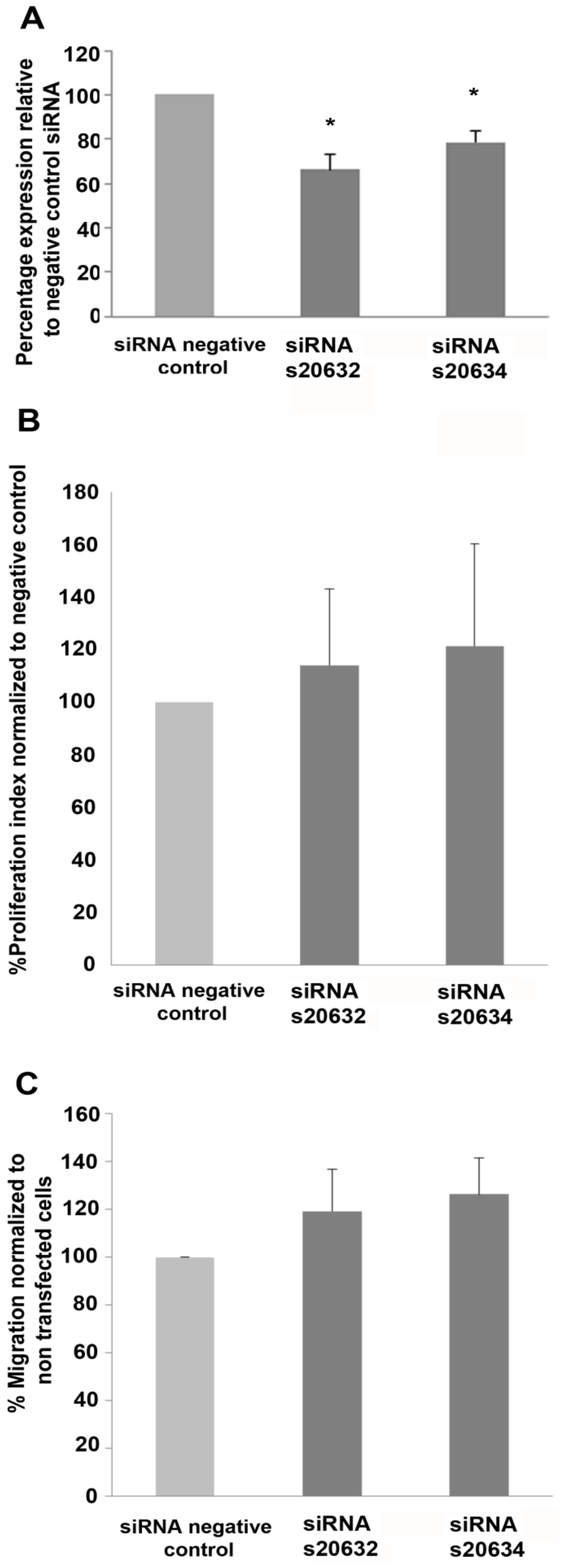

Supplementary Fig. S5. HYOU1 does not affect CB ECFC-derived cell proliferation and migratory abilities

A. Western blot quantification of HYOU1 protein levels in CB ECFC-derived cells following transfection of 2 different siRNAs for HYOU1, each at a final concentration of 10 nM. Target protein expression was normalised to tubulin expression in each sample. Results show target gene protein levels as a percentage of the negative control siRNA-transfected sample for each concentration. (*$p \leq 0.05$ one-way ANOVA, Dunnett's multiple comparison).

B. The effect of HYOU1 siRNA knockdown on the proliferative ability of CB ECFC-derived cells under the specified culture conditions. Cells treated with either a negative control siRNA, HYOU1 s20632 siRNA, or HYOU1 s20634 siRNA at a final concentration of 10nM were subjected to a CyQuant proliferation assay at 72 hr to quantify cell number. Fluorescence values were normalised to those obtained on the day of transfection for each sample to obtain a proliferation index for each condition ($p>0.05$; one-way ANOVA, Dunnett's multiple comparison).

C. The effect of HYOU1 siRNA knockdown on the migration of CB ECFC-derived cells. Cells treated with either a negative control siRNA, HYOU1 s20632 siRNA, or HYOU1 s20634 siRNA at a final concentration of 10nM were subjected to a transwell assay for 5 hr towards 10% EGM-2 growth media following 48 hr post-transfection. Migration values were normalised to those obtained for the negative control siRNA transfected samples ($p>0.05$; one-way ANOVA, Dunnett's multiple comparison).

1 2 3 4

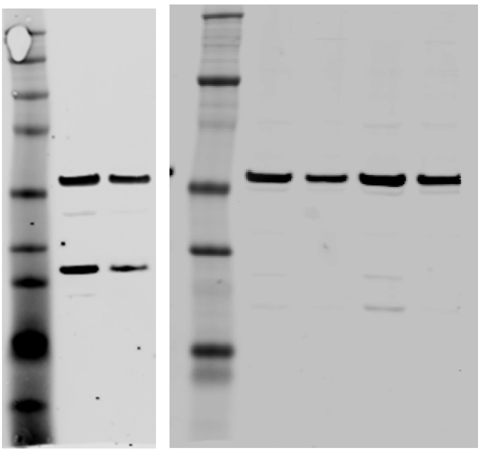

HMGB1 HYOU1

Supplementary Fig. S6. Full length blots from Figure 5A. Lane 1, 3- Control CB ECFC-derived cells, Lane 2,4 - Mimic 193a-3p treated CB ECFC-derived cells (Tubulin upper band, HMGB1/HYOU1 lower band)

1 2 3

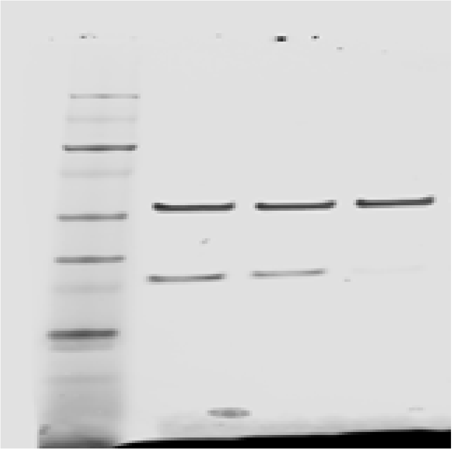

Supplementary Fig. S7. Full length blots from Figure 6A. CB ECFC-derived cells transfected with siRNA negative control (Lane 1), siRNA 20254 (Lane 2), siRNA 20255 (Lane 3)

Supplementary Table S1. Target prediction for miRNA-193a-3p based on the proteins identified from the proteome array

		Targetscan	miRanda	miRDB	miRWalk	RNAhybrid	Total
		**Verion 5.1**	**August 2010 release**	**April 2009**	**Performed 5.11.2014**	**Version 2.1**	
hsa-miR-193a-3p	CPSF6	Y	Y	N	Y	Y	4
hsa-miR-193a-3p	DOK6	N	Y	N	Y	N	2
hsa-miR-193a-3p	EIF4G2	N	N	N	Y	N	1
hsa-miR-193a-3p	HMGB1	Y	Y	N	N	Y	3
hsa-miR-193a-3p	HYOU1	Y	Y	N	Y	N	3
hsa-miR-193a-3p	NT5E	Y	Y	Y	Y	Y	5
hsa-miR-193a-3p	PCBP1	N	N	N	N	Y	1
hsa-miR-193a-3p	PTTG1IP	Y	Y	N	Y	Y	4
hsa-miR-193a-3p	SNRPE	N	N	N	N	Y	1
hsa-miR-193a-3p	STMN1	Y	N	Y	Y	Y	4
hsa-miR-193a-3p	TNPO1	N	N	N	Y	Y	2
hsa-miR-193a-3p	XPNPEP1	Y	Y	Y	Y	Y	5

Twelve proteins were predicted to target miR-193a-3p using 5 target prediction sites:- Targetscan, miRanda, miRDB, miRWalk and RNAhybrid
